# A cryogenic 14‐channel ^13^C receiver array for 3T human head imaging

**DOI:** 10.1002/mrm.29508

**Published:** 2022-11-02

**Authors:** Wenjun Wang, Juan Diego Sánchez‐Heredia, Rie Beck Olin, Esben Søvsø Szocska Hansen, Christoffer Laustsen, Vitaliy Zhurbenko, Jan Henrik Ardenkjær‐Larsen

**Affiliations:** ^1^ National Space Institute Technical University of Denmark Kongens Lyngby Denmark; ^2^ Department of Health Technology Technical University of Denmark Kongens Lyngby Denmark; ^3^ MR Research Center, Department of Clinical Medicine Aarhus University Aarhus Denmark

**Keywords:** carbon imaging, cryogenic coil, head coil, MRI array

## Abstract

**Purpose:**

This article presents a novel 14‐channel receive‐only array for ^13^C human head imaging at 3 T that explores the SNR gain by operating at cryogenic temperature cooled by liquid nitrogen.

**Methods:**

Cryostats are developed to evaluate single‐coil bench SNR performance and cool the 14‐channel array with liquid nitrogen while having enough thermal insulation between the coils and the sample. The temperature distribution for the coil array is measured. Circuits are adapted to the −189°C environment and implemented in the 14‐channel array. ^13^C images are acquired with the array at cryogenic and room temperature in a 3T scanner.

**Results:**

Compared with room temperature, the array at cryogenic temperature provides 27%–168% SNR improvement over all voxels and 47% SNR improvement near the image center. The measurements show a decrease of the element noise correlation at cryogenic temperature.

**Conclusion:**

It is demonstrated that higher SNR can be achieved by cryogenically cooling the 14‐channel array. A cryogenic array suitable for clinical imaging can be further developed on the array proposed. The cryogenic coil array is most likely suited for scenarios in which high SNR deep in a head and decent SNR on the periphery are required.

## 1 INTRODUCTION

^13^C MRI, due to its ability to visualize the metabolic process inside human bodies invisible to ^1^H MRI, promises to be a valuable tool for characterizing cancer and monitoring early response to cancer treatment.^1^, ^2^ For example, decreased conversion rate of [1‐^13^C]pyruvate to [1‐^13^C]lactate can reveal early response to treatment by X‐ray irradiation much before decrease of tumor volume.^3^ In MRI, high SNR is required for high‐quality images. For ^13^C imaging, the demand for high SNR is more peremptory than for ^1^H imaging, as the signal of ^13^C is severely limited by the 1.1% natural abundance and low gyromagnetic ratio^4^ (γ[C13]≈0.25γ[H1]). On the one hand, the MR signal of ^13^C can be substantially increased by hyperpolarization^5^; on the other hand, coils can be carefully designed to decrease noise and increase reception sensitivity. Because the major source of noise in MRI is thermal noise, whose RMS voltage is proportional to the square root of the temperature and the square root of the equivalent resistance,^6^ a way to achieve higher SNR is to reduce noise on receiving coils by cooling coils to cryogenic temperature. This approach is useful when the noise from the coils dominates over noise from the imaged object.^7^ For coils of diameter small than approximately 40 mm with zero coil‐to‐sample distance, at the resonance frequency 32.13 MHz (Larmor frequency of ^13^C at 3 T) and room temperature, the thermal noise of the coils dominates over the sample noise.^7^, ^8^ Therefore, cryogenic coils may be particularly suitable for a ^13^C receive coil array for 3T human brain imaging.

The development of cryogenic coils began in the 1980s.^9^ Most cryogenic coils up to present are built for ^1^H high‐field spectroscopy,^9^ imaging for small objects,^10^ and ^1^H imaging at low field such as 0.17 T^11^ and 0.21 T.^12^ Apart from ^1^H, cryogenic coils for ^19^F imaging at 9.4 T^13^ and ^13^C imaging at 3 T^14^ in rodents have also been developed. Despite different resonant frequencies, in all these situations, the noise from the coils dominates over noise from the sample. For ^1^H imaging, the SNR gain* typically ranges from 1.7 fold^15^ to 4.0 fold^16^; an impressive 9.8‐fold gain has also been reported.^17^ For ^13^C mouse imaging, an SNR gain of near 2‐fold is reported.^14^ Human in vivo imaging by cryogenic coils is mostly confined to skin^8^, ^18^ and fingers^19^ at 1.5 T. In vivo imaging of the hand,^20^ the wrist,^21^ the brain,^22^ and the knee^22^, ^23^ by cryogenic coils has been performed at low field strength below 0.21 T.

Various cryostats have been developed to cool coils. A simple setup is to immerse coils in liquid nitrogen^15^, ^24^ or helium^9^, ^16^ directly. Simple as it is, the seething liquid inside the cryostat can cause thermal instability.^25^ More sophisticated designs have coils mounted on heat‐conductive substrates like sapphire^26^ and alumina,^14^, ^27^ which are put in direct contact with liquid^20^ or gas^10^, ^28^, ^29^ coolants. Closed‐cycle coolers like Gifford‐McMahon refrigerators,^30^ Joule‐Thomson machines,^31^, ^32^ and pulse‐tube coolers^31^, ^33^ can also be used to cool coils, even cryogen‐free.^32^, ^33^

Phased arrays^34^ are nowadays widely used for signal reception because of their ability to reduce sample noise and yield high SNR on surfaces and deeper inside objects^35^ and to enable image acceleration through parallel imaging.^36^ Phased arrays have been developed for imaging ^1^H^37^ and nonproton nuclei^38^ like ^13^C^39^ and ^31^P^40^. However, experiments with cryogenic coil arrays are sparse. For ^1^H imaging, two‐channel^41^, ^42^ and four‐channel^43^ cryogenic arrays have been reported with SNR gain from 2.0 fold^41^, ^43^ to 2.4 fold^42^. The two‐channel cryogenic array by Kwok et al is used to image a human hand.^42^ To the authors' best knowledge, no cryogenic array for ^13^C imaging has been reported so far. Exploring the MR performance of a cryogenic coil array for ^13^C imaging is therefore of particular interest. A cryogenic coil array for clinical ^13^C imaging will be worthwhile if substantial SNR improvement can be found on a prototype array.

In this article, a prototype, proof‐of‐concept 14‐channel cryogenic coil array for ^13^C head imaging at 3 T is described. The main goal is to explore the imaging performance of a cryogenic array without aiming at a specific SNR gain in advance. Two cryostats are developed for evaluating single coils and carrying the 14‐channel array. The electronic circuits are adapted for cryogenic temperature. Images are acquired with the array and a human‐sized head phantom with both the array at room temperature and cooled by liquid nitrogen.

## 2 METHODS

### 2.1 Cryostats

Two cryostats are designed: one for evaluating the performance of a single element of the array, and the other for imaging using the 14‐channel array. Both cryostats are RF‐transparent, use nonmagnetic materials, and are compatible with 3T MRI. Because the cryogenic array is a proof of concept, simple structures that can offer sufficiently long cooling and decent thermal insulation between coils and human heads are chosen for the cryostats. Nonetheless, coils ought not to be directly immersed in liquid nitrogen, as it easily damages electronic components. Thus, in our designs, the coils are mounted on alumina, which, with the other end in direct contact with liquid nitrogen or the vessel containing liquid nitrogen, cools down the coils. These simple designs allow various coils and arrays to be mounted on the cryostats and evaluated for their cryogenic performance reliably.

#### 2.1.1 Cryostat #1 for benchtop measurements

**FIGURE 1:**
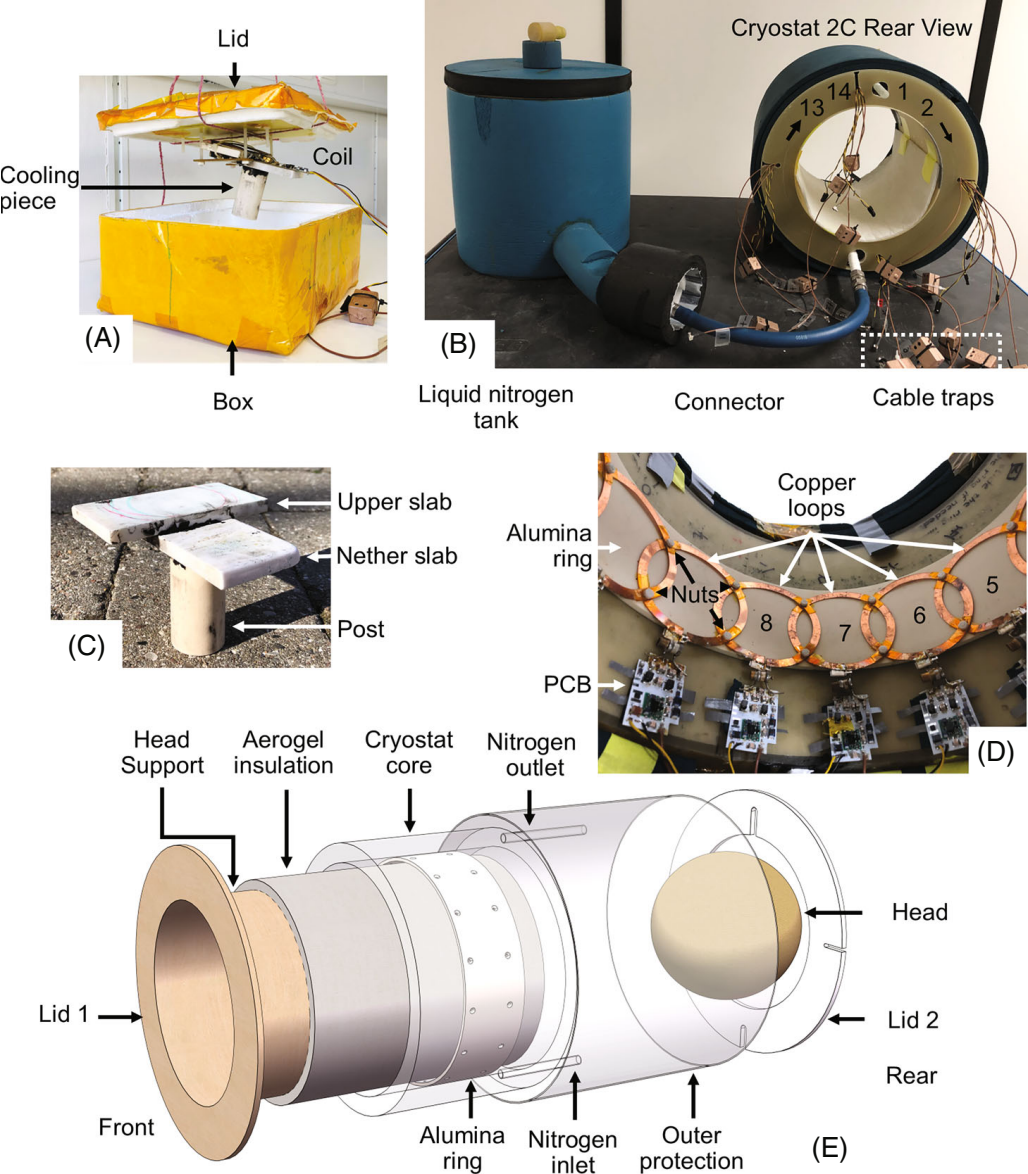
A, Cryostat #1, a Styrofoam box of 213 × 255 × 125 mm size. B, Cryostat #2 assembly, which consists of a liquid nitrogen tank, a tube connector, and cryostat 2C. The copper‐clad small boxes with two “eyes” each are cable traps. Coils mounted inside cryostat 2C are labeled 1–14 as marked. C, The alumina piece on which coils are mounted inside cryostat 1. D, The inside of the cryostat 2C. Copper rings are fixed by zirconia (ZrO_2_) screws and nuts on an alumina ring (coils are numbered 5–8). E, Cryostat 2C. Liquid nitrogen flows through nitrogen inlet and exhausts through the nitrogen outlet. Holes are drilled on lid 2 to let wires and cables pass through. A head is drawn as a ball for simplicity of graph drawing. Abbreviation: PCB, printed circuit board

Cryostat #1 for evaluating single coils is an oblong Styrofoam box of size 213 × 255 × 125 mm as shown in Figure 1A. The full volume inside is 1.6 L. The lid is 15.5‐mm thick. Two 100 × 50 × 8 mm^3^ alumina (Al_2_O_3_) slabs and one alumina post of 30‐mm diameter and 60‐mm height are glued by Loctite STYCAST 2850FT (Henkel AG & Co., Düsseldorf, Germany) to form an alumina cooling piece shown in Figure 1C.

During experiments, a coil is first mounted onto the ceramic piece, which is later attached by nylon screws and nuts onto the cryostat, as shown in Figure 1A. Then, the cryostat is filled with liquid nitrogen. Next, the lid closes the cryostat. The temperature of the upper slab of the alumina piece in cryostat #1 is measured by Pt100 sensors and recorded by a model 218 temperature monitor (Lake Shore Cryotronics, Westerville, OH, USA). Cryogenic thermal contact grease (M&I Materials, Manchester, United Kingdom) is applied on Pt100 sensors to ensure good thermal contact.

#### 2.1.2 Cryostat #2 for imaging with a 14‐channel array

Cryostat #2 for carrying the 14‐channel array consists of a liquid nitrogen tank, a cryostat 2C, and a tube connector, as shown in Figure 1B. Outer surfaces of all parts of the cryostat are clad by ArmaFlex Ultima (Armacell, Mamer, Luxembourg) for good thermal insulation. The liquid nitrogen tank is a hollow cylinder of glass fiber that opens on the top with outer diameter 305 mm, inner diameter 200 mm, height 300 mm, and full volume 9.4 L. The tube connector is PB‐8 (Swagelok, Solon, OH, USA). More adaptors from Swagelok are used for joining the tube with the liquid nitrogen tank and cryostat 2C. These adaptors are made of stainless steels of little magnetism. The complete assembly of the cryostat in Figure 1E consists of the following parts:

1. Two lids of glass fiber, of which lid 2 has holes drilled on to let cables and wires through;
2. An outer protection of glass fiber for protection with outer diameter 365 mm, thickness 2.0 mm, and length 300 mm;
3. A cryostat core of glass fiber for holding liquid nitrogen with outer diameter 328 mm, inner diameter 280 mm, and length 210 mm;
4. A head support of glass fiber for placing a head with outer diameter 243 mm, thickness 2.0 mm, and length 300 mm;
5. An alumina ring for coil cooling with outer diameter 280 mm, thickness 4.5 mm, length 80 mm, mounted between the inner part and the cryostat core; and
6. An Aerogel insulation part made of two layers of 6‐mm Thermal Wrap Aerogel blanket (Cabot, Boston, MA, USA).

**FIGURE 2:**
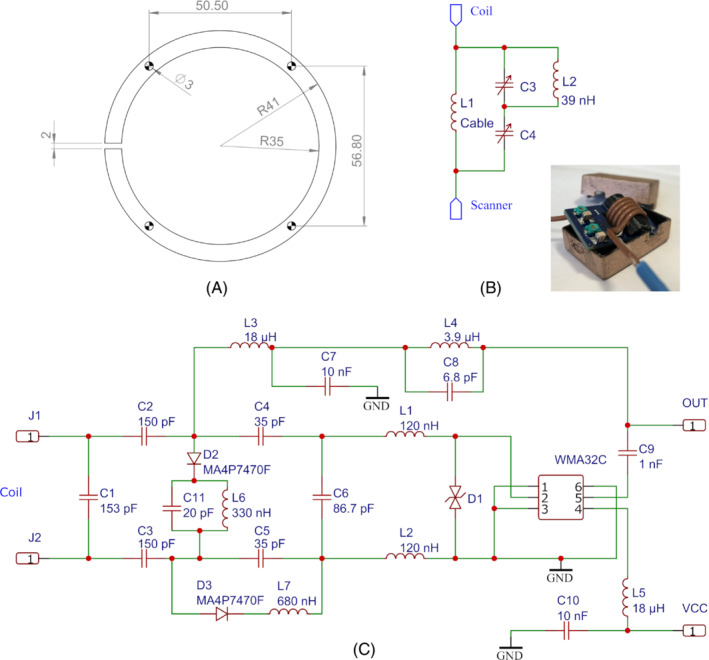
A, Coil dimensions. The four holes on the ring are for fixing coil on an alumina ring in cryostat 2C shown in Figure 1B,D. B, The schematic of the cable trap. L_1_ is formed by a coaxial cable. The inset is a photograph of a cable trap with encasement. C, The biasing and matching circuit for preamplifier WMA32C. C_1_–C_6_ and L_1_–L_2_ comprise the matching circuit. D_2_, L_6_, C_11_, D_3_, and L_7_ comprise the active decoupling circuit

The dimensions of cryostat 2C, especially the head support and the alumina ring, are chosen to fit an average human head with the largest diameter of approximately 225 mm while keeping necessary distance between the head and cryogenic coils for thermal insulation. Because of the proof‐of‐concept nature of the cryogenic array in this article, only one alumina ring is installed to demonstrate the effect of cryogenic cooling on one slice. There are 15 pairs of through‐holes on the alumina ring in Figure 1D,E for ISO M3 countersunk zirconia (ZrO_2_) screws for fixing the copper coils. The holes in a pair are separated by 50.5‐mm center‐to‐center distance. Each pair is separated by 56.8‐mm center‐to‐center distance, in line with the coil dimensions shown in Figure 2A. Based on the dimensions, 14 coils as described in section 2.2 can be installed on the ring. The coil loops are labeled 1–14 clockwise from the top of cryostat, as shown in Figure 1B. When the coil loops are installed, layers of electrical insulator are inserted between overlapping loops.

For use, the liquid nitrogen tank of cryostat #2 is filled full. After the liquid nitrogen drops to half, the liquid nitrogen tank is filled full again, and not replenished afterward. The temperature of the coil loops in cryostat #2 is also measured by Pt100 sensors and recorded by the model 218 temperature monitor. To keep the temperature of the head above 0°C during imaging, one more layer of 3‐mm Aerogel blanket is padded on cryostat 2C. This makes the coil‐to‐head separation 18 mm. Hot air can be blown into cryostat 2C to increase the temperature and improve patient comfort.

For simplicity of nomenclature, the term “cryostat #2” refers to the whole setup in Figure 1B or Cryostat 2C shown by Figure 1E hereafter, depending on the context.

### 2.2 ^13^C coils

As noise in MRI primarily comes from the coil and the sample—which is a head in this case—the dimensions of coils are chosen so that, at room temperature, the coil's thermal noise dominates, and at cryogenic temperature, the coil's noise at least loses dominance. If the coils are too large, the sample's thermal noise always dominates; if the coils are too small, the coil's thermal noise always dominates. Therefore, there is a range of coil size in which cryogenic cooling turns out to be the most beneficial.

Based on this reasoning, all coils are receive‐only, single‐turn copper loops, of which the dimensions are chosen as outer diameter 82.0 mm, inner diameter 70.0 mm, and thickness 0.45 mm.^44^ These coil dimensions were used earlier in a 14‐channel array for 3T ^13^C imaging (coil #6 in Olin et al^45^) with a coil‐to‐sample distance of 2 mm. It was found that these dimensions, together with the coil‐to‐head separation of 15.5–18 mm, satisfy the principle of noise dominance transition described previously. Four holes of diameter 3 mm separated by 50.5 × 56.8 mm are drilled on coils for mechanical fixing. The coil inductance is 147.5 nH at 32.13 MHz. The coils are matched to a WMA32C (WanTCom, Chanhassen, MN, USA) preamplifier. The matching circuit is designed as proposed by Reykowski et al^46^ and Roemer et al.^34^ For better decoupling, the coils are matched to 180 Ω at cryogenic temperature rather than the typical 50 Ω.^47^, ^48^ The effect can be seen in Supporting Information Table S1, where, at room temperature, matching to 180 Ω gives higher coil Q than matching to 50 Ω. Matching coils to 180 Ω at room temperature gives a 1.16‐dB room‐temperature preamplifier noise, which is 0.48 dB higher than the minimum noise figure. For good noise performance, the matching circuit is built by low‐loss, high‐Q PPI 1111C capacitors (Passive Plus, Huntington, NY, USA) and Coilcraft Midi inductors (Coilcraft, Cary, IL, USA). A PIN diode pair MML4401‐GM3 (Microsemi, Aliso Viejo, CA, USA) is installed in front of the preamplifier for extra protection during transmission phase. The output of the preamplifier is connected to a 50‐Ω coaxial cable. To suppress common‐mode current on outer shields of cables,^35^, ^49^ for each cable output, a doubly tuned cable trap is constructed^50^ and shielded by copper‐painted polymer boxes 3D printed on a Prusa i3 MK3S+ (Prusa Research, Prague, Czechia).

The bench SNRs of the coils are measured by enclosing a coil and a small transmit loop in a CMW‐Z10 shielded box (Rohde & Schwarz, Munich, Germany). The coil is loaded by a phantom of saltwater emulating a human head.^44^ Signal is excited by an SMC100A signal generator (Rohde & Schwarz) and received by an E4440A spectrum analyzer (Keysight Technologies, Santa Rosa, CA, USA). Coil Q and preamplifier decoupling are measured by a double‐loop probe connected to a ZNL3 vector network analyzer.^34^, ^46^ The matching network is adjusted so that the SNR peak and the local decoupling extremum are located at 32.13 MHz at cryogenic temperature.

To determine the optimal coil overlap at cryogenic temperature, two coils are matched to 50‐Ω cables connected to a ZNL3 vector network analyzer (Rohde & Schwarz). The optimal overlap is identified when the $\left|S_{21}\right|$ or $\left|S_{12}\right|$ reaches the minimum at 32.13 MHz. Several pairs of coils are also printed on flexible printed circuit boards and overlapped at different distances to quantify the optimal overlap. It is found that, at the optimal overlap, the center‐to‐center coil distance at cryogenic temperature is a little shorter than at room temperature, but the difference is less than 0.25 mm.

**FIGURE 3:**
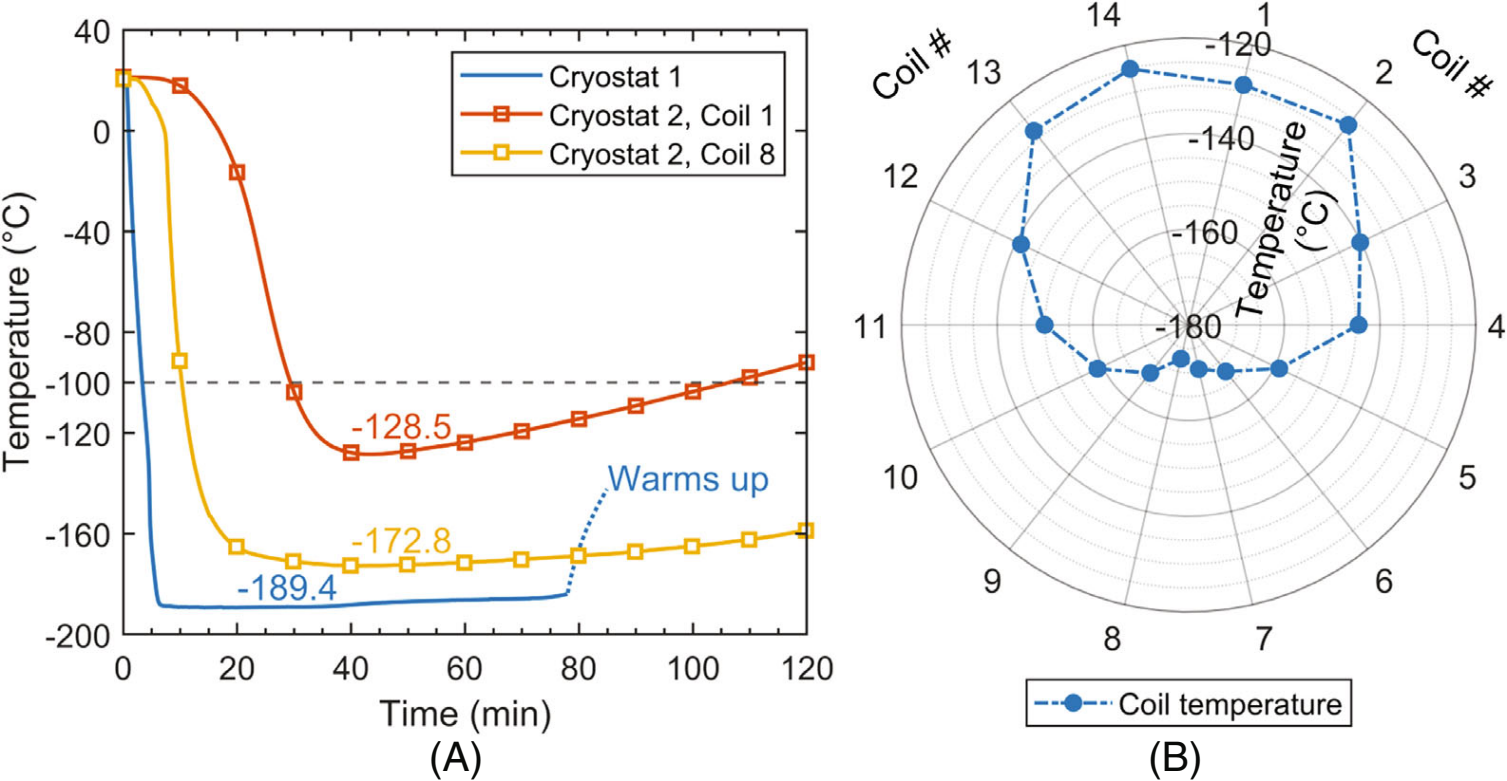
A, Temperature of cryostat #1, and coils 1 and 8 of cryostat 2 versus time. Cryostat #1 needs 6.5 min to cool and sustains temperature for approximately 1 h 10 min. Cryostat #2 needs 30 min to cool and remains useable for approximately 1 h 10 min if no liquid nitrogen is replenished. B, Steady‐state temperature distribution of coils in cryostat #2 if liquid nitrogen is not replenished. Coils 1 and 14 stand on the top of cryostat #2. Coils 7 and 8 lie at the bottom. The lowest temperature (−172.8°C) is found on coil 8. The highest temperature (−125.1°C) is found on coil 14

The DC current of the WMA32C amplifier drops after cooling. The lowest DC current observed is 3.9 mA, at which WMA32C malfunctions, as shown by Supporting Information Figure S1. To solve the problem, circuits of WMA32C amplifiers on channels 5–10 (refer to Figure 3B) are modified to increase the cryogenic DC current to 9–10 mA.

### 2.3 Image acquisition

**FIGURE 4:**
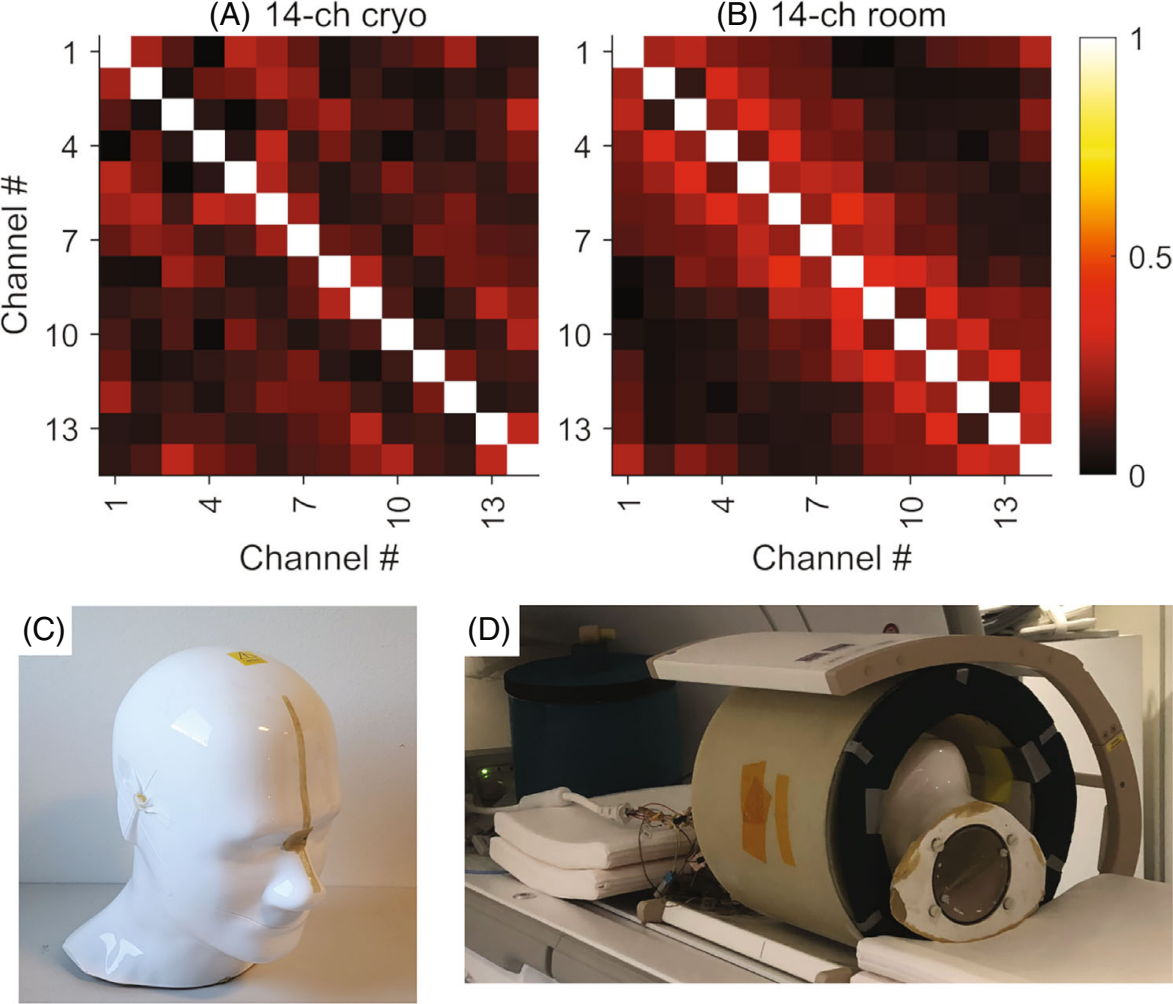
The noise correlation of the 14‐channel array at cryogenic (A) and room temperature (B). C, The head‐shaped phantom. D, The experiment setup. At cryogenic temperature, the average absolute values of the off‐diagonal elements of the correlation matrix is $\bar{\left|\rho \right|}=0.118$ (range: 0.004≤|ρ|≤0.289). At room temperature, $\bar{\left|\rho \right|}=0.147$, 0.006≤|ρ|≤0.424. These correlation values are also listed in Table 2

The 14‐channel array is tested by a human head–sized phantom. A head‐shaped Specific Anthropomorphic Mannequin phantom (IXB‐030; IndexSAR, Horsham, United Kingdom) with 300‐mm top‐to‐bottom height, 225‐mm maximum outer perimeter, as shown in Figure 4C, is used. The phantom is filled with 99.8% ethylene glycol solution of natural abundance ^13^C (Sigma Aldrich, St. Louis, MO, USA) and 17 g/L NaCl for tissue loss emulation. The concentration of NaCl is chosen to match tissue conductivity at 32 MHz of 0.66 S/m.^45^ The phantom is scanned using a clinical 3T MR scanner (MR750; GE Healthcare, Waukesha, WI, USA). The cryogenic array mounted in cryostat #2 (described in section 2.1.2) is used for signal reception. A clamshell ^13^C transmit coil (RAPID Biomedical, Rimpar, Germany) is used for transmission. The experimental setup is shown in Figure 4D.

A quality assurance protocol^51^ described by Olin et al^45^ is used to estimate array SNR. Signal is acquired with a non‐refocused CSI sequence with center frequency at ethylene glycol central peak resonance. The sequence is scanned in the axial plane with FOV = 360 × 360 mm, slice thickness = 20 mm, matrix size = 24 × 24. The nominal spatial resolution is 15 × 15 × 20 mm. Other sequence parameters are soft pulse excitation, nominal flip angle = 70°, TR = 1000 ms, spectral bandwidth = 5000 Hz, and FID points = 1024. The total scan time is 9 min 36 s. The sequence is run with TE = 3.06 ms. A separate noise acquisition follows with TR = 1000 ms, spectral bandwidth = 5000 Hz, FID points = 1024, and flip angle = 0°. The noise‐equivalent bandwidth factor is 0.845.^45^

Images are first acquired at room temperature. Afterward, liquid N_2_ is poured into cryostat #2 as described in section 2.1.2. Image acquisition begins 40 min after cooling to ensure coils have reached their nominal temperature.

A geometry factor map, or *g‐factor map*, is derived from the data set of the CSI sequence. The k‐space is undersampled retrospectively with acceleration rates of 2 and 3 in the right–left direction. The 1/g maps are constructed by the SENSE method^36^ and can be interpreted as maps of SNR reduction due to parallel imaging. Noise covariance matrices are calculated from the data set of noise‐only acquisition and converted to noise correlation matrices.

## 3 RESULTS

### 3.1 Cryostat temperature profiles

The curves of temperature versus time for cryostats #1 and #2 are shown in Figure 3A. At time 0 min, liquid nitrogen is poured, and cooling begins. The alumina piece of cryostat #1 reaches −189°C in approximately 6.5 min and lasts about 1 h 10 min without replenishment of liquid nitrogen. For cryostat #2, after 30 min of cooling, the temperature of coil loop 8 reaches −170°C and the temperature of coil loop 1 reaches −104°C. If liquid nitrogen is not replenished, the temperature of coil 1 keeps below −100°C for approximately 1 h 10 min. Benchtop SNR characterization takes approximately 5 min, so cryostat #1 suffices for that. Imaging takes about 10 min, and Cryostat #2 suffices as well.

The steady‐state temperature of coil loops on cryostat #2 is drawn in Figure 3B. The temperature profile is symmetric about the vertical line, indicating reliable thermal contact between the alumina ring and the cryostat core (refer to Figure 1E). The lowest temperature (−172.8°C) is found on loop 8. The highest temperature (−125.1°C) is found on coil 14. The temperature difference is 47.7°C. By blowing 50°C air from the rear of cryostat #2 flush with lid 2 (Figure 2B,E), the temperature at which the phantom touches the head support can stay above 18°C.

### 3.2 Coil Q and benchtop output SNR

The Q values of a single coil and coil 8 inside the 14‐channel array is indicated in Table 1. When measuring Q, a coil is connected to its matching network, but the preamplifier is disconnected. In the presence of a phantom, the bench cryogenic SNR is measured to be 1.35 times the room‐temperature SNR. The measured SNR as a function of frequency for a single coil at cryogenic temperature and room temperature for the range 32.13±0.50MHz is shown in Figure 6F with the room‐temperature SNR at 32.13 MHz normalized to 100. The maximum SNR of a room‐temperature single coil is 108 at (32.13‐0.14)MHz. The maximum SNR of a cryogenic single coil is 136 at 32.13 MHz. The maximum cryogenic SNR is 1.26 times the maximal room‐temperature SNR.

**TABLE 1:** Q of a single coil and Q of coil 8 in the 14‐channel array

		Coil Q, single	Coil Q, in array
Cryogenic	Loaded	274	207
	Unloaded	687	223
Room	Loaded	208	148
	Unloaded	330	162

*Note*: Coil Q is measured when a coil is connected to its matching network without preamplifier connected. For a single coil, “cryogenic” refers to −189°C. For coil 8 in the 14‐channel array, “cryogenic” refers to −172°C. “Loaded” means phantom is present. “Unloaded” means phantom is not present.

### 3.3 Image SNR and g‐factor

**FIGURE 5:**
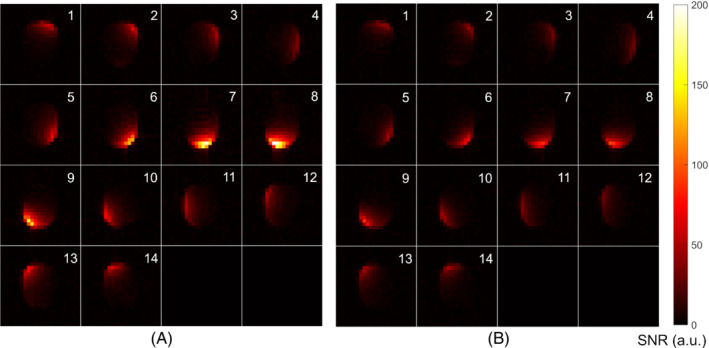
Individual coil SNR profiles of the array at cryogenic temperature (A) and at room temperature (B). The signal has an arbitrary unit. Numbers 1–14 on the upper‐right corner of each picture mark the coil numbers that correspond to Figure 3B. The SNR increases at cryogenic temperature for all channels

The noise‐correlation matrices of the 14‐channel array at cryogenic and room temperatures are shown in Figure 4A,B, respectively. The correlation data are listed Table 2. Both the maximum and average off‐diagonal noise correlation decrease at cryogenic temperature. The SNR maps of the individual channels at cryogenic and room temperature are shown in Figure 5. The SNR increases at cryogenic temperature for all channels.

**TABLE 2:** Maximum, central, and average SNR of the 14‐channel array at cryogenic and room temperature, and the 14‐channel array in “Reference” (Olin et al^45^); and maximum, average, and minimum off‐diagonal noise correlation

	SNR			Noise correlation		
	Max.	Central	Avg.	Max.	Avg.	Min.
Cryogenic	276	47.4	86.3	0.289	0.118	0.004
Room temperature	128	32.2	50.1	0.424	0.147	0.006
Reference	252	40	–	0.218	0.053	0.003

*Note*: Noise correlation of Reference is extracted from Figure 6 of Olin et al.^45^ The head‐to‐coil separation of “cryogenic” and “room temperature” is 18 mm. The head‐to‐coil separation of “Reference” is 2 mm.

**FIGURE 6:**
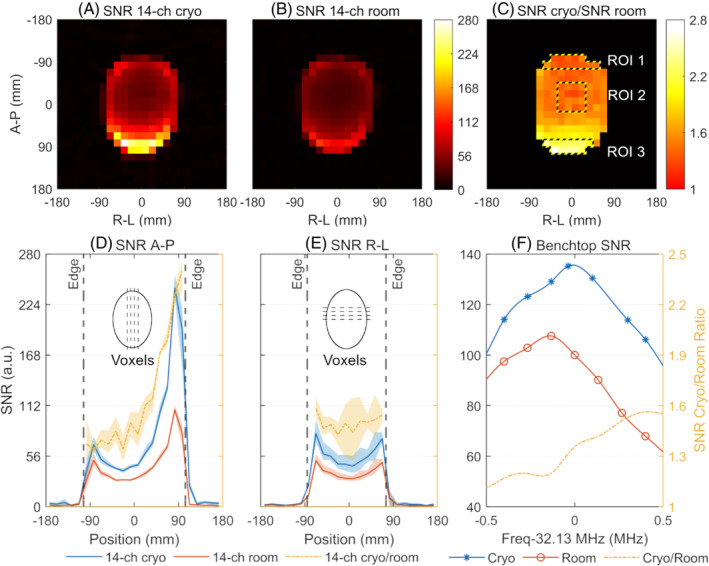
A, The SNR map of the cryogenic coil array after coil combination. B, The SNR map of the coil array at room temperature. C, The map of SNR gain, calculated by the array SNR at cryogenic temperature divided by the array SNR at room temperature. The statistical data of regions of interest (ROIs) 1, 2, and 3 that correspond to top, central, and bottom areas are found in Table 3. D, The SNR profile of the centermost four columns of the image in anterior–posterior direction (A‐P). E, The SNR profile of the centermost four rows of the image in right–left direction (R‐L). The SNR follows axes on the left. The SNR gain follows axes on the right. Shaded stripes depict the corresponding ranges of SNR and SNR gain. Solid lines delineate the average SNR or SNR gain. The maximum, central, and average SNR values are listed in Table 2. The highest, lowest, central, and average SNR gain at cryogenic temperature over room temperature are 2.68×, 1.27×, 1.47×, and 1.64×, respectively. F, The benchtop SNR of a single coil at cryogenic and room temperature versus frequency. The abscissa is f−32.13MHz. The benchtop room‐temperature SNR at 32.13 MHz is set to 100. Parts (D)–(F) share the same scale of SNR cryo/room ratio

The SNR maps for the combined images from the array at cryogenic and room temperature are shown in Figure 6A,B, respectively. The 14‐channel array at cryogenic temperature yields higher SNR than at room temperature. Maximal, central, and average SNR data are listed in Table 2. The map of SNR gain calculated by the array SNR at cryogenic temperature divided by the SNR at room temperature is shown in Figure 6C. The SNR profiles along the centermost four lines in anterior–posterior (A‐P) and right–left (R‐L) directions are shown in Figure 6D,E respectively, along with the SNR gain at cryogenic temperature over room temperature.

The highest SNR gain is 2.68 × on voxel A‐P −37.5 mm, R‐L 97.5 mm. The lowest SNR gain is 1.27 × on voxel A‐P 7.5 mm, R‐L −22.5 mm. The average SNR gain of all voxels in Figure 6C is 1.64 times. The average SNR gain on the top, at the center, and at the bottom are found in Table 3.

**TABLE 3:** Ratio of cryogenic SNR to room‐temperature SNR, including benchtop SNR of a single‐coil, image SNR in ROIs 1, 2, and 3 in Figure 6C

		Image		
SNR Cr/Rm	Benchtop, single coil	ROI 1	ROI 2	ROI 3
Min.		1.27	1.27	2.05
Avg.	1.35	1.39	1.47	2.33
Max.		1.60	1.65	2.68

*Note*: The benchtop SNR ratio has a single value 1.35.

Abbreviation: SNR Cr/Rm, ratio of cryogenic SNR to room‐temperature SNR.

**FIGURE 7:**
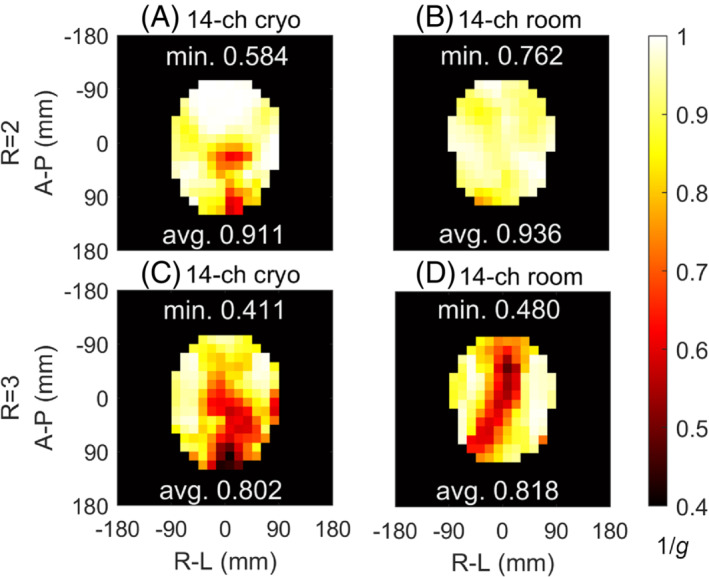
Retrospective SENSE 1/g factor maps of the coil array at cryogenic temperature with acceleration rate 2 (A), at room temperature with acceleration rate 2 (B), at cryogenic temperature with acceleration rate 3 (C), and at room temperature with acceleration rate 3 (D). Acceleration occurs in the R‐L direction. Abbreviations: Avg., average 1/g factor; Min., minimum 1/g factor

The 1/g maps of the 14‐channel array operating at cryogenic and room temperature with 2‐times acceleration in the R‐L direction are shown in Figure 7A,B, respectively. The 1/g maps at cryogenic and room temperature with 3‐times acceleration in the R‐L direction are shown in Figure 7C,D, respectively. The average and minimum 1/g values are shown in Figure 7. The 14‐channel coil at cryogenic temperature has similar average 1/g values compared with at room temperature, but lower minimum 1/g, corresponding to a greater parallel‐imaging SNR reduction.

## 4 DISCUSSION

### 4.1 Temperature of cryostat #2

As described in section 2.1.2, liquid nitrogen is not replenished after some point, which results in the 47.7°C temperature difference between the coils on the top (coils 1 and 14) and at the bottom (coils 7 and 8). As the liquid nitrogen level drops, the coils on the top gradually loses thermal contact with liquid nitrogen. While alumina still transfers heat between the bottom and the top, thermal dissipation eventually causes the temperature on the top to rise. To thoroughly cool the coils, devices that automatically replenish liquid nitrogen could be installed on cryostat #2. In practice, however, the safety issues of placing a liquid nitrogen supply near an MR scanner can be complicated.

Using the coil Q values in Table 1, it can be estimated that the sample contributes 59.1 mΩ of resistance to the coil; the loop contributes 43.3 mΩ at −189°C, and contributes 90.2 mΩ at room temperature (22°C). Because copper resistivity is linear with temperature down to −243.1°C (30 K),^25^ it can be estimated that, below −100°C, the loop will contribute less than 51.6% of the total coil resistance. In the experiment, the lowest temperature on coil 14 is −125.1°C, and images are acquired around the time when the temperature on coil 14 is near its minimum. In this case, the loop contributes less than 49.3% of the total coil resistance. Therefore, the imaging acquisition setup at cryogenic temperature ensures that loop noise does not dominate.

### 4.2 Signal‐to‐noise ratio and noise correlation

The SNR gain of central voxels in the A‐P direction distributes less evenly than the SNR gain of central voxels in the R‐L direction. There are three reasons for this: (1) The phantom is not a cylinder, so the bottom of phantom is much closer to coils, improving the peripheral SNR at the bottom; (2) the temperature of the cryogenic coils at the bottom is 48°C lower than the coils on the top, further increasing the SNR at the bottom of the phantom; and (3) there is no coil on the very top of cryostat #2, as Supporting Information Figure S2 shows, which yields insufficient SNR on the top of the phantom. Despite the unevenness of the temperature, the higher SNR and the decreased noise correlation of the cryogenic array demonstrates its good performance.

From Figure 6F, it can be concluded that the improvement of image SNR at cryogenic temperature over SNR at room temperature is not caused solely by drift of SNR peak after cooling. To account for the drift of the SNR maximum, the SNR improvement can be corrected by a factor of 1/1.076, which yields image SNR gain ranging from to 1.18 times to 2.49 times with an average of 1.52 times. Therefore, there is “authentic” SNR gain on all voxels in the images.

The noise correlation of the array at cryogenic temperature is lower than at room temperature. This is likely because, first, the coil overlap is optimized for cryogenic operation, so that coils decouple more at cryogenic temperature, which is suggested by the Q improvement from 162 to 223 after cooling, as indicated in Table 1; second, at cryogenic temperature, low temperature and lower coil resistance may decrease noise covariance,^52^, ^53^ which may decrease noise correlation.

The SNR and noise correlation of the 14‐channel array can be compared against a room‐temperature 14‐channel array described by Olin et al,^45^ labeled coil #6 therein. The coil is benchmarked with the same phantom as in this paper, and the coil‐to‐head separation is 2 mm. Its maximum SNR is 252, and the central SNR is 40, as presented in Table 2 of Olin et al^45^ (also in Table 2 of this paper, row “Ref”). Both the maximum and central SNR are slightly lower than the maximum (276) and central SNR (43.0) of the cryogenic 14‐channel array in this paper. The noise correlation of the cryogenic 14‐channel array is higher than the 14‐channel array in Olin et al,^45^ but not significantly higher, as Table 2 indicates. These data suggest that the cryogenic array, despite wider coil‐to‐head separation, yields SNR performance at least on par with the 14‐channel array of Olin et al.^45^ Higher SNR may be achieved by smaller coil‐to‐head separation, which, however, requires better insulation of cryostat #2, as discussed in section 4.1.

### 4.3 1/*g* factors

The cryogenic array has lower minimum 1/*g* factors compared with the room‐temperature array. At 2‐times acceleration, min1/g=0.584 for the cryogenic array and 0.762 for the array at room temperature. However, this greater SNR reduction is less than the SNR gain shown in Figure 6, which means that the SNR for the cryogenic array will still be higher than that of the room‐temperature array after parallel imaging. Consider that, at 2‐times acceleration $\left(\min1/g_{\text{cryo}}\right):\left(\min1/g_{\text{room}}\right)=0.767$, the minimum SNR gain without acceleration is 1.27, so that the *worst‐case SNR gain* under acceleration is 0.767×1.27=0.974, very close to 1. This indicates that the SNR will at least not drop at cryogenic temperature. At 3‐times acceleration, $\left(\min1/g_{\text{cryo}}\right):\left(\min1/g_{\text{room}}\right)=0.856$, the *worst‐case SNR gain* under acceleration is 0.856×1.27=1.09, which also indicates that SNR will not drop at cryogenic temperature. It should be noted that the worst‐case SNR gain is calculated by $\min1/g_{\text{cryo}}$, $\min1/g_{\text{room}}$, and minimum SNR gain on different voxels; therefore, the actual SNR gain is far higher than the bounds above.

A possible reason for the lower 1/g factors of the cryogenic array, despite lower noise correlations, is a change in effective geometry of the array due to uneven cooling. When the array is cooled down, the temperature effect is spatially asymmetric (Figure 3B), leading to different variances of the coil channels. This can be seen in Supporting Information Figure S3, in which the noise covariances at room temperature and cryogenic temperature follow different patterns, with lower variances of channels 6, 8, and 9 in particular, corresponding to channels at the bottom of the array. The exact *g*‐factors are influenced by both the geometry of the imaged object and the applied undersampling strategy relative to the coil geometry. Different *g*‐factors are therefore expected with different methods. Thus, while the cryogenic array has lower 1/g factors in this case, this might not be true for other cases.

### 4.4 Overall evaluation and future directions

On the particular experiment setup in section 2.3, despite problems like temperature unevenness (section 3.1), thick thermal insulation (section 2.1.2), but low temperature on the sample (section 3.1), the 14‐channel array yields 27%–168% higher SNR than at room temperature, with the central SNR improvement being 47%. As demonstrated in section 4.2, on the periphery of an object, the SNR of the cryogenic coil is not significantly higher than a 14‐channel array at room temperature with 2 mm of space between coils and sample. Unique to the cryogenic array is the SNR improvement at the image center, which results from reduced thermal noise power of coils. For the cryogenic coil to be applied to clinical human imaging, the following aspects can be improved:

- Liquid nitrogen can be fed from the top rather than the bottom, which will likely improve temperature evenness;
- Warm air can be blown into cryostat #2 during use to warm the head. Better thermal insulation and air‐tight cryostat design can prevent warm air from heating coils; and
- Thinner thermal insulation can be devised, which can improve SNR on the periphery of a head. However, evacuation is likely needed.^54^

In general, it is likely that the 14‐channel cryogenic coil array is most suited for scenarios in which higher SNR than room‐temperature arrays is desired deep in the head, while peripheral SNR on par with room‐temperature arrays is also required.

## 5 CONCLUSIONS

A prototype 14‐channel array operable at cryogenic temperature is made for ^13^C human head imaging at 3 T. A cryostat is made for the 14‐channel array to mount on, which shows reasonably good thermal distribution. The array at cryogenic temperature has lower noise correlation and higher SNR than at room temperature. Highest *g*‐factor, and therefore SNR reduction, is found at cryogenic temperature, but not high enough to erase the SNR gain compared with the array at room temperature. The cryogenic array features high SNR improvement at the image center deep inside a head, and decent SNR on the periphery. A cryogenic array for clinical human head imaging can be built on the prototype array with a better cryostat design and better thermal insulation.

## Supporting information


**Figure S1** Transmission path gain versus DC current at room and cryogenic temperature for WMA32C. At 4.0‐mA DC bias current, WMA32C does not function well, so the bias circuit of WMA32C is changed to provide 9–10‐mA DC quiescent current

**Figure S2** Coils mounted on the alumina ring. Screws and nuts are not drawn. There is a vacancy on the top. The alumina ring is drawn transparent for display

**Figure S3** The absolute values of noise covariance of the array at cryogenic temperature (A) and room temperature (B). C, The noise variance of each channel in the array. The noise variance at cryogenic temperature follows a different pattern from the room‐temperature variance, which implies altered effective geometry of the array

**Table S1** Individual room‐temperature unloaded coil Q in the 14‐channel array when matching to 50 Ω and 180 Ω. For coil 7, matching to 180 Ω yields higher room‐temperature Q than 50 Ω‐matching cryogenic Q

## ENDNOTE

* We use “gain of a” to mean a×, and “improvement of a” to mean (1+a)×.
