# Supplementary figures and images for: A cryogenic 14‐channel 13C receiver array for 3T human head imaging
Source: Magn Reson Med. 2022 Nov 2;89(3):1265–77. doi: 10.1002/mrm.29508 (PMC10092528; doi:10.1002/mrm.29508)

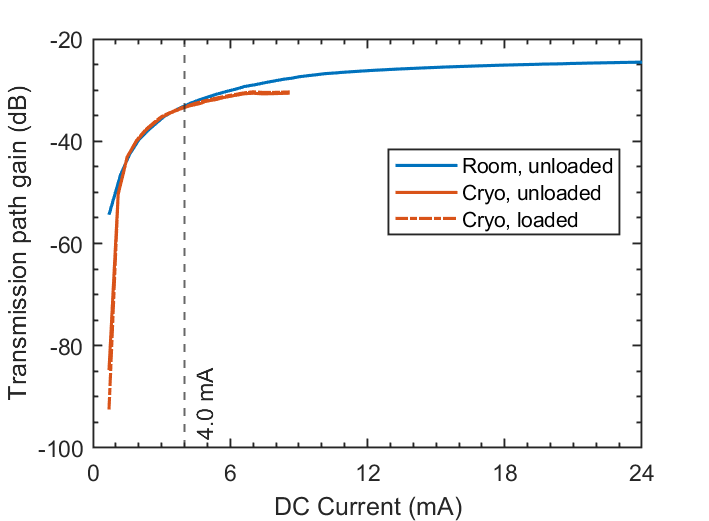

Supplement: Supplementary file 1 — Figure S1 Transmission path gain versus DC current at room and cryogenic temperature for WMA32C. At 4.0‐mA DC bias current, WMA32C does not function well, so the bias circuit of WMA32C is changed to provide 9–10‐mA DC quiescent current [file MRM-89-1265-s004.tif]

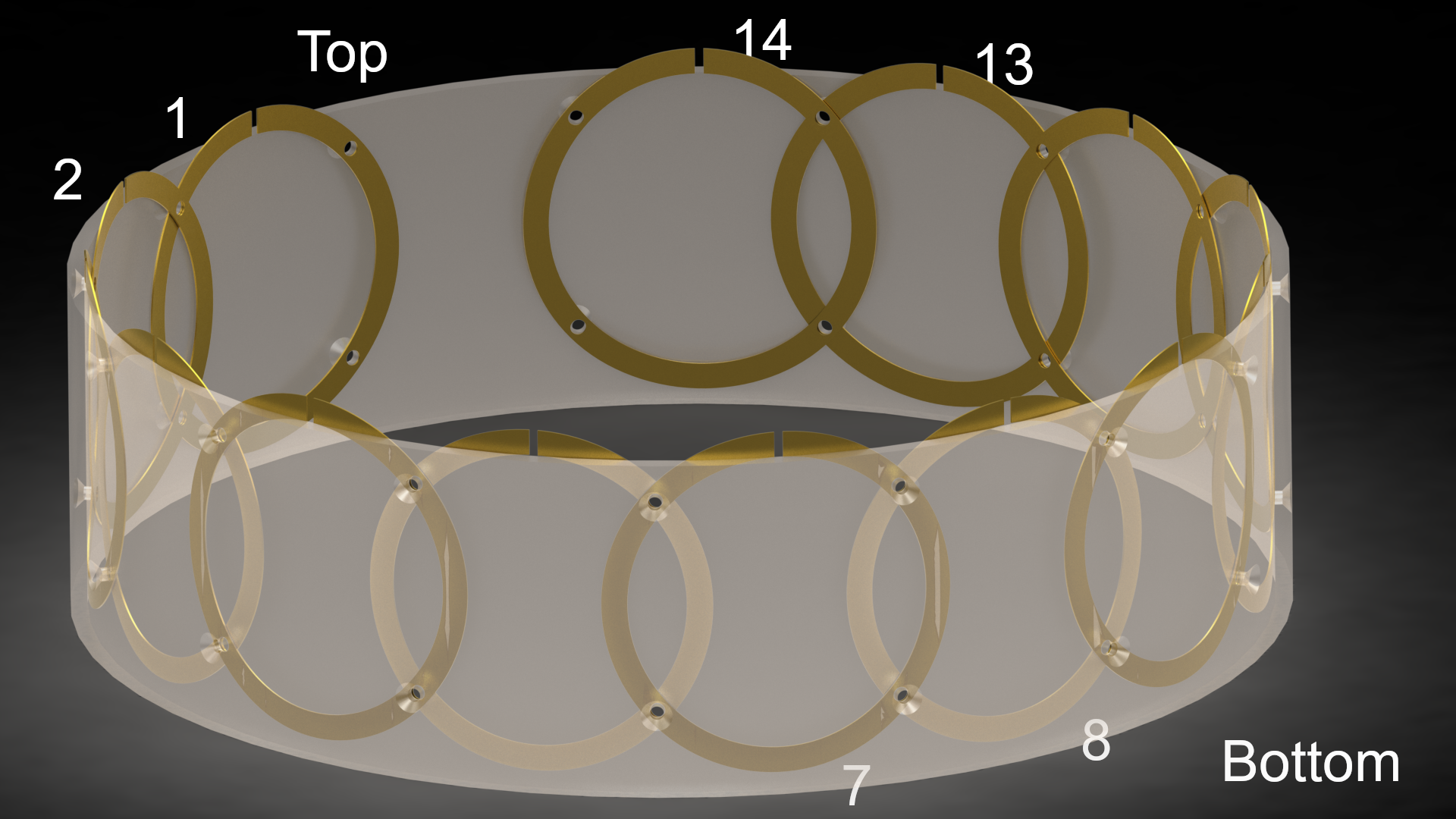

Supplement: Supplementary file 2 — Figure S2 Coils mounted on the alumina ring. Screws and nuts are not drawn. There is a vacancy on the top. The alumina ring is drawn transparent for display [file MRM-89-1265-s003.tif]

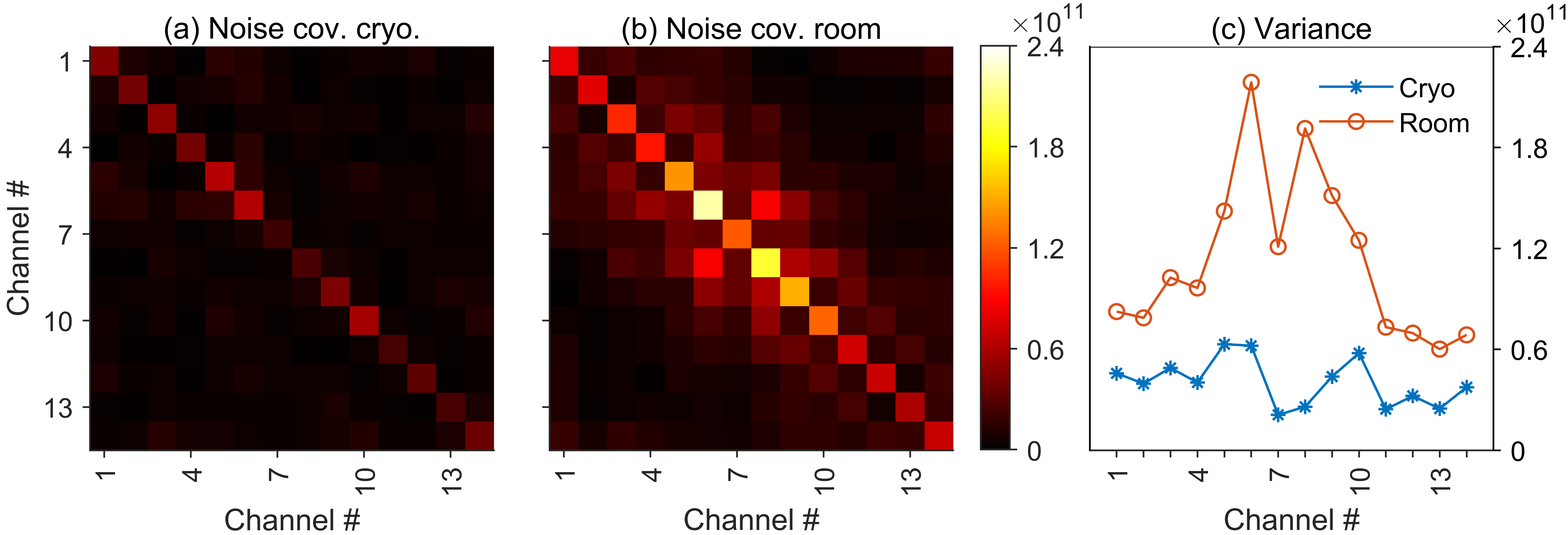

Supplement: Supplementary file 3 — Figure S3 The absolute values of noise covariance of the array at cryogenic temperature (A) and room temperature (B). C, The noise variance of each channel in the array. The noise variance at cryogenic temperature follows a different pattern from the room‐temperature variance, which implies altered effective geometry of the array [file MRM-89-1265-s001.tif]
