# Supplementary material for: A cryogenic 14‐channel 13C receiver array for 3T human head imaging
Source: Magn Reson Med. 2022 Nov 2;89(3):1265–77. doi: 10.1002/mrm.29508 (PMC10092528; doi:10.1002/mrm.29508)
Supplement: Supplementary file 4 — Table S1 Individual room‐temperature unloaded coil Q in the 14‐channel array when matching to 50 Ω and 180 Ω. For coil 7, matching to 180 Ω yields higher room‐temperature Q than 50 Ω‐matching cryogenic Q [file MRM-89-1265-s002.pdf]

SUPPORTING INFORMATION FIGURES AND TABLES

Supporting Information Table S1. Individual room-temperature unloaded coil Q in the 14-channel array when matching to 50 Ω and 180 Ω. For coil 7, matching to 180 Ω yields higher room-temperature Q than 50 Ω-matching cryogenic Q

Channel #	To 50 Ω	To 180 Ω
1	104	175
2	127	155
3	100	177
4	100	167
5	102	174
6	110	174
7	111[i]	174
8	77.7	162[ii]
9	96.3	157
10	106	163
11	106	161
12	105	180
13	135	216
14	104	177

[i] Cryogenic value 137

[ii] Cryogenic value 223
